# Association Between Neutrophil-to-Lymphocyte Ratio and Sepsis Severity in ICU Patients

**DOI:** 10.7759/cureus.71687

**Published:** 2024-10-17

**Authors:** Saad Binliaquat, Urooj Arshad, Muhammad Ali Shahid, Ahmed Yar Khan, Yamin Htet, Muhammad Umair Mazhar, Abdul Eizad Asif, Tayyab Mumtaz Khan

**Affiliations:** 1 Emergency Medicine, Royal Derby Hospital, Derby, GBR; 2 Emergency Medicine, Wrightington, Wigan and Leigh NHS Foundation Trust, Wigan, GBR; 3 Acute Medicine, University Hospitals Coventry and Warwickshire NHS Trust, Coventry, GBR; 4 Stroke Medicine, University Hospitals Coventry and Warwickshire NHS Trust, Coventry, GBR; 5 Internal Medicine, Shalamar Medical and Dental College, Lahore, PAK; 6 Orthopedic Surgery, Rawalpindi Medical University, Rawalpindi, PAK

**Keywords:** association, care, intensive, lymphocyte, neutrophil, patients, ratio, sepsis, severity, unit

## Abstract

Background

Sepsis is a potentially fatal condition that necessitates prompt identification and assessment of its severity for effective management. However, evaluating sepsis severity using the Sequential Organ Failure Assessment (SOFA) and Acute Physiology and Chronic Health Evaluation (APACHE II) scores can be complex and costly. This study aimed to assess the association between neutrophil-to-lymphocyte ratio (NLR) and sepsis severity, as well as the role of NLR as a predictive indicator of sepsis severity in ICU patients.

Methods

This cross-sectional study was conducted among 180 ICU-admitted patients at Benazir Bhutto Hospital (BBH) in Rawalpindi, Pakistan, from January 2022 to January 2023. Participants were enrolled using defined inclusion and exclusion criteria along with consecutive sampling. Following ethical approval and informed consent, data were collected using a self-structured form. The study population was divided into three groups based on sepsis severity, which was assessed via the SOFA score. Data analysis was performed using IBM SPSS Statistics for Windows, Version 25.0 (Released 2017; IBM Corp., Armonk, NY, USA) through chi-squared tests, one-way ANOVA, Pearson’s correlation, and a simple linear regression model, with a significance threshold set at p < 0.05.

Results

In the study population of 180 patients, the frequencies of sepsis, severe sepsis, and septic shock were 69 (38.34%), 86 (47.78%), and 25 (13.88%), respectively. Significant variations were observed among the three study groups in the means of the PaO2/FiO2 ratio, mean arterial pressure, Glasgow Coma Scale score, total bilirubin level, serum creatinine level, platelet count, SOFA score, neutrophil count, lymphocyte count, and NLR (p < 0.05). Pearson’s correlation analysis indicated a strong positive correlation between the NLR and SOFA score, with a correlation coefficient (r) of 0.80 and significance at p < 0.001. Furthermore, linear regression analysis identified NLR as a significant predictor of sepsis severity, with a beta coefficient (β) of 3.55 and a 95% CI of 1.92-5.60 (p < 0.001).

Conclusions

In the current study, a positive and significant correlation was found between the NLR and the severity of sepsis. Higher NLR values were associated with increased SOFA scores, indicating a greater severity of sepsis. This study supports the use of NLR as a complementary and cost-effective tool for the early detection of high-risk patients with sepsis, facilitating timely interventions and improving outcomes, particularly in under-resourced healthcare settings.

## Introduction

Sepsis is a life-threatening condition that arises from a dysregulated response to infection, leading to injury to the body’s tissues and organs. It presents along a clinical spectrum, ranging from mild bacteremia to severe sepsis and ultimately resulting in septic shock [1]. According to the Third International Consensus Definition for Sepsis and Septic Shock (Sepsis-3), sepsis is characterized as a potentially fatal condition where a dysregulated host response to infection results in life-threatening organ dysfunction. Septic shock represents a severe subset of sepsis, marked by significant circulatory, cellular, and metabolic impairments that substantially increase the risk of mortality [2]. The manifestation of sepsis can vary based on the origin of the infection and its severity. Common nonspecific symptoms include malaise, fever or hypothermia, tachycardia, tachypnea, low blood pressure, altered mental status, and reduced urine output [3,4]. Primary causes of sepsis include respiratory tract infections, intra-abdominal infections, bloodstream infections, urinary tract infections, and skin infections [5-7].

Sepsis poses a staggering global healthcare burden, affecting over 30 million people annually and resulting in approximately six million deaths worldwide [6]. In the United States, sepsis cases exceed 1.7 million each year, with a mortality rate of 15% [6]. Similarly, in Pakistan, sepsis accounts for up to 45% of ICU admissions, with a mortality rate of 37% [7]. The economic toll of sepsis is significant, with estimated annual costs exceeding $24 billion in the United States. Furthermore, survivors often experience lasting consequences, including cognitive, physical, and emotional impairments, as well as chronic diseases, which substantially impact their quality of life [6,8].

Early detection, timely antibiotic administration, fluid resuscitation, vasopressor support, and organ support (such as mechanical ventilation) are critical components of sepsis management [9,10]. However, diagnosing sepsis presents challenges due to nonspecific initial symptoms, the lack of a clear biomarker, overreliance on the complex Sequential Organ Failure Assessment (SOFA) scoring system, difficulties in differentiating sepsis from non-infectious systemic inflammatory response syndrome, and the low sensitivity of blood cultures. Various scoring systems, including SOFA and Acute Physiology and Chronic Health Evaluation (APACHE II), have been used to assess the severity and prognosis of sepsis, but their application is complicated by a multitude of factors affecting sepsis outcomes - such as age, comorbidities, immunosuppression, infection site, causative pathogens, antibiotic resistance, fluid adequacy, and nutritional status. Moreover, calculating scores from these systems often necessitates several investigations, leading to increased costs as well [11-15]. Therefore, there is an urgent need for simple, effective, and affordable predictors of sepsis severity and prognosis to reduce the global burden of this condition.

In recent years, various biomarkers have emerged as valuable tools for predicting sepsis severity and guiding clinical decision-making. Among these, the neutrophil-to-lymphocyte ratio (NLR) has gained attention due to its simplicity, cost-effectiveness, and potential to reflect the balance between pro-inflammatory and anti-inflammatory responses in sepsis. NLR is calculated by dividing the neutrophil count by the lymphocyte count, providing a quantitative measure of the immune response [15]. Several studies have explored the relationship between NLR and sepsis severity, yielding promising results; however, the majority of these investigations have been conducted in developed countries. The association between NLR and sepsis remains understudied in developing countries [16-21].

Despite the growing recognition of NLR’s importance in sepsis management, there is a lack of research in Pakistan focusing on its utility in assessing sepsis severity among ICU patients. By examining the predictive capacity of NLR for sepsis severity, this research aims to contribute to the development of simple, cost-effective biomarkers for the early identification and management of sepsis, particularly in resource-limited settings.

## Materials and methods

Study design and study population

This cross-sectional study was conducted in the ICU of Benazir Bhutto Hospital (BBH) in Rawalpindi, Pakistan, over one year, from January 2022 to January 2023. A total of 180 patients diagnosed with sepsis were enrolled through consecutive sampling, surpassing the calculated minimum sample size of 150. This sample size was determined based on a prevalence of sepsis of 10.90% from a study by Baykara et al., with a 5% margin of error and a 95% CI [4]. Strict inclusion and exclusion criteria were implemented to ensure the validity of the study.

Inclusion and exclusion criteria

The study included patients of any gender, aged 18 years and older, who had complete laboratory results and medical records, a known source of infection, and a confirmed diagnosis of sepsis. These patients were admitted to either medical or surgical ICUs within the past 24 hours. Conversely, patients were excluded from the study if they had a history of antibiotic treatment within the last 24 hours, were pregnant, had congenital heart disease, blood disorders, malnutrition, chronic inflammatory diseases, malignancy, a history of steroid use within the three months preceding ICU admission, or had undergone chemotherapy or radiotherapy within the last six weeks. Additionally, patients with do-not-resuscitate orders (as per Pakistan Medical and Dental Council guidelines) and those who did not provide consent for participation in the research were also excluded.

Ethics

This study received ethical approval from the Ethics Review Board at BBH, Rawalpindi, Pakistan (approval number BBH.ERB.283.221). Additionally, written informed consent was obtained from each participant after thoroughly explaining the study’s objectives and procedures, ensuring their complete understanding and voluntary participation.

Primary outcome and secondary outcomes

The primary objective of this study was to investigate the association between serum NLR and the severity of sepsis, as measured by SOFA scores, in ICU patients. In addition to this primary outcome, the study pursued three secondary objectives. First, it aimed to identify the common causes of sepsis among ICU patients. Second, NLR values were compared across three distinct patient groups: those with sepsis, severe sepsis, and septic shock. Lastly, the study assessed the predictive capacity of NLR for the progression of sepsis severity, including the development of organ failure, to evaluate its potential utility as a prognostic marker.

Sepsis and severity of sepsis

Sepsis was defined according to the criteria established by the American College of Chest Physicians and the Society of Critical Care Medicine. It is characterized as life-threatening organ dysfunction resulting from a dysregulated host response to infection. For practical clinical application, organ dysfunction is considered present when there is an increase of 2 or more points in the SOFA score [2]. The SOFA score was utilized to evaluate organ dysfunction across six systems: respiratory (PaO2/FiO2 ratio), cardiovascular (hypotension and vasopressor requirement), hepatic (bilirubin level), coagulation (platelet count), renal (creatinine level and urine output), and neurological (Glasgow Coma Scale (GCS) score). The SOFA score ranges from 0 to 24 and categorizes patients into three groups: sepsis (3-5), severe sepsis (6-11), and septic shock (12-24). While this scoring system has been widely used globally, Cronbach’s alpha was calculated to validate the SOFA score system within our population, yielding a score of 0.81 for 50 responses, indicating excellent reliability and internal consistency [4,11].

NLR calculation

The NLR was calculated from peripheral blood samples by dividing the neutrophil count by the lymphocyte count. In healthy adults, the NLR typically ranges from 0.78 to 3.53 [22].

Sample collection for the required investigations

Blood samples from all patients were collected by registered nurses in accordance with hospital protocols that comply with the standards set by the College of American Pathologists. These samples were analyzed to determine various blood parameters, including complete blood count, total bilirubin levels (0.2-1.3 mg/dL), creatinine levels (0.7-1.3 mg/dL for men and 0.6-1.1 mg/dL for women), and the PaO2/FiO2 ratio (≥400 mmHg) through arterial blood gas analysis.

Data collection

Data collection for this study was conducted using a specially designed questionnaire comprising three distinct sections. The first section focused on demographic information (age and gender), medical history, and physical examination findings, including heart rate, blood pressure/mean arterial pressure, respiratory rate, temperature, and GCS score. The second section addressed the laboratory investigation reports. The third section utilized data from the initial two sections to calculate the SOFA score and the NLR value.

Data analysis

Statistical analysis was conducted using IBM SPSS Statistics for Windows, Version 25.0 (Released 2017; IBM Corp., Armonk, NY, USA). Quantitative data were summarized as mean ± SD, while qualitative data were expressed as frequencies and percentages. One-way ANOVA and chi-squared tests were utilized to compare numerical and nominal variables across the three study groups, respectively. Pearson’s correlation analysis examined the relationship between the NLR and SOFA scores. Additionally, a linear regression model assessed the predictive value of NLR for SOFA scores. A p-value of less than 0.05 was considered statistically significant.

## Results

Frequency of sepsis, severe sepsis, and septic shock and etiology

Among the 180 patients, sepsis was identified in 69 (38.34%), severe sepsis in 86 (47.78%), and septic shock in 25 (13.88%) patients. The primary causes of sepsis in this population included respiratory tract infections (98 patients, 54.44%), intra-abdominal infections (40 patients, 22.22%), urinary tract infections (22 patients, 12.23%), skin infections (14 patients, 7.77%), brain infections (four patients, 2.23%), and bone infections (two patients, 1.12%).

Comparison of demographic and clinical characteristics among the three study groups

Table 1 presents the demographic and clinical details of the study population, highlighting significant differences among the three study groups (sepsis, severe sepsis, and septic shock) in several key variables, including total bilirubin level, serum creatinine level, platelet count, mean arterial pressure, PaO2/FiO2 ratio, GCS score, SOFA score, neutrophil count, lymphocyte count, and NLR, all with p < 0.05. In contrast, no statistically significant differences were observed in age and gender distribution across the three groups, with p > 0.05, indicating an even distribution of these demographic factors.

**Table 1:** Characteristics of the study population with one-way ANOVA and chi-squared test analysis p < 0.05: statistically significant In the last column, p-values with the sign of (+) are of ANOVA tests, while p-values with the sign of (*) are of the chi-square test. FiO2: fraction of inspired oxygen; GCS: Glasgow Coma Scale; NLR: neutrophil-to-lymphocyte ratio; PaO2: partial pressure of arterial oxygen; SOFA: Sequential Organ Failure Assessment

Variables, Patients with sepsis (N = 180)		Expression of variables	Severity of sepsis			One-way ANOVA test/chi-square test
			Sepsis group, n = 69 (38.34%)	Severe sepsis group, n = 86 (47.78%)	Septic shock group, n = 25 (13.88%)	p-values
Gender	Male, n (%)	106 (58.89%)	38 (55.10%)	51 (59.30%)	18 (72.00%)	0.08^*^
	Female, n (%)	74 (41.11%)	31 (44.90%)	35 (40.70%)	7 (28.00%)	
Age (years), mean ± SD		58.44 ± 12.12	61.18 ± 11.42	62.06 ± 10.22	64.25 ± 12.41	0.06^+^
Platelets count (×10^9^/L), mean ± SD		178.99 ± 22.61	140.12 ± 34.12	118.87 ± 21.25	101.33 ± 12.23	0.002^+^
Total bilirubin level (mg/dL), mean ± SD		2.90 ± 1.47	1.30 ± 0.46	3.90 ± 1.78	4.93 ± 2.89	0.001^+^
Serum creatinine level (mg/dL), mean ± SD		2.89 ± 2.12	2.16 ± 0.39	3.45 ± 2.44	4.41 ± 1.80	0.001^+^
Mean arterial pressure (mmHg), mean ± SD		89.99 ± 7.88	85.40 ± 7.82	77.67 ± 8.44	69.46 ± 14.30	0.001^+^
PaO2/FiO2 ratio (mmHg), mean ± SD		390.49 ± 49.40	318.17 ± 42.76	295.11 ± 40.12	234.37 ± 46.21	0.002^+^
GCS score, mean ± SD		12.08 ± 2.29	12.42 ± 2.51	10.69 ± 4.41	7.01 ± 5.39	0.003^+^
SOFA score, mean ± SD		7.04 ± 4.24	5.10 ± 2.14	9.80 ± 1.46	15.22 ± 3.56	0.001^+^
Neutrophils count (×10^9^/L), mean ± SD		15.34 ± 9.23	16.72 ± 8.18	17.82 ± 11.31	19.73 ± 10.54	0.001^+^
Lymphocytes count (×10^9^/L), m± SD		0.91 ± 0.58	0.85 ± 0.50	0.79 ± 0.44	0.74 ± 0.30	0.001^+^
NLR, mean ± SD		15.86 ± 7.24	17.65 ± 6.44	20.55 ± 7.44	24.60 ± 6.57	0.002^+^

Correlation between NLR and the severity of sepsis

Table 2 demonstrates a statistically significant positive correlation between NLR values and the severity of sepsis, as assessed through Pearson’s correlation analysis in the study population. This correlation suggests that as NLR values increase, SOFA scores also tend to rise, indicating a direct relationship between NLR and the severity of sepsis.

**Table 2:** Correlation between NLR and the severity of sepsis in the study population p < 0.05: statistically significant NLR, neutrophil-to-lymphocyte ratio

Variables, N = 180	Severity of sepsis			One-way ANOVA test	Pearson’s correlation	
	Sepsis group	Severe sepsis group	Septic shock group	p-value	Correlation coefficient (r)	p-value
SOFA score, mean ± SD	5.10 ± 2.14	9.80 ± 1.46	15.22 ± 3.56	0.001^+^	0.8	0.001
NLR, mean ± SD	17.65 ± 6.44	20.55 ± 7.44	23.60 ± 6.57	0.002^+^		

NLR as a prognostic marker of the severity of sepsis

Table 3 illustrates that the simple linear regression model demonstrated an excellent fit (R² = 0.81, p < 0.0001), revealing a statistically significant positive relationship between NLR values and SOFA scores. The positive beta coefficient indicates that higher NLR values are associated with increased SOFA scores, reflecting greater sepsis severity.

**Table 3:** Assessment of the predictive value of NLR for the severity of sepsis through a simple linear regression model p < 0.05: statistically significant The R2 value was 0.81 (81.00%). The regression model was significant as the p-value of the F test was <0.000 (significant). NLR: neutrophil-to-lymphocyte ratio

Variable	Unstandardized regression coefficient (β)	95% CI	p-value
NLR	3.55	1.92-5.60	0.001

## Discussion

Sepsis is a life-threatening condition arising from the body’s dysregulated response to infection and poses a significant global healthcare burden. Its management is highly complex, and a quicker and more effective response to sepsis increases the likelihood of survival. In the current study, we obtained valuable data on the association between the NLR and the severity of sepsis in patients in the ICU. We also assessed variations in the means of key parameters among three study groups: patients with sepsis, severe sepsis, and septic shock. These variables included the PaO2/FiO2 ratio, mean arterial pressure, GCS score, total bilirubin level, serum creatinine level, platelet count, SOFA score, neutrophil count, lymphocyte count, and NLR.

In our study population, 69 patients (38.34%) had sepsis, 86 patients (47.78%) had severe sepsis, and 25 patients (13.88%) had septic shock. A comparable Chinese study reported similar results, with 137 patients (41.14%) having sepsis, 149 patients (44.74%) having severe sepsis, and 47 patients (14.11%) having septic shock [18]. In contrast, a Turkish study observed lower frequencies: 163 patients (10.90%) with sepsis, 260 patients (17.30%) with severe sepsis, and 203 patients (13.50%) with septic shock [4]. The variability in sepsis severity across different countries may be attributed to factors such as environmental conditions, comorbidities, underlying medical conditions, microbial epidemiology, infection control methods, and healthcare infrastructure.

The primary causes of sepsis in our population were respiratory tract infections, followed by intra-abdominal infections, urinary tract infections, skin infections, brain infections, and bone infections. Literature supports these findings, with several investigations demonstrating similar etiologies of sepsis [1-4].

Regarding demographics, our study found that men predominated among patients with increased sepsis severity. Additionally, there was an observed trend of increasing severity of sepsis with advancing age. These demographic patterns align with findings from various studies that have presented similar observations regarding the incidence of sepsis [19,20].

Among the three study groups - patients with sepsis, severe sepsis, and septic shock - statistically significant variations in key parameters, including the PaO2/FiO2 ratio, mean arterial pressure, GCS score, total bilirubin level, serum creatinine level, platelet count, SOFA score, neutrophil count, lymphocyte count, and NLR, were noted (p < 0.05). Previous studies employing the SOFA score and NLR to evaluate sepsis severity have corroborated our results regarding differences in the means of these key parameters [1,18-20].

Our study found a significant positive association between NLR and sepsis severity; patients with greater severity of sepsis exhibited higher NLR values. This key finding is consistent with multiple studies conducted in various regions worldwide. For instance, a study from Greece confirmed NLR as a significant and cost-effective predictor of sepsis severity [15]. A meta-analysis also demonstrated that NLR increases with sepsis severity and that high NLR predicts poor prognosis among sepsis patients [16]. Additionally, research from Sweden established a reliable correlation between NLR and sepsis, indicating NLR as a biomarker for both bacteremia and severe sepsis [17]. A Chinese study similarly reported that as NLR increases, so does the severity of sepsis [18]. Consistent findings from the Netherlands showed that patients with higher NLR experienced more severe sepsis than those with lower NLR [19]. Moreover, a study from Romania noted differences in NLR values among patients with varying severities of sepsis [20]. An Indian study further indicated that NLR can serve as both a diagnostic and prognostic factor for sepsis [21]. These results support the use of NLR as a reliable and practical biomarker for assessing sepsis severity.

The mechanism underlying NLR’s association with sepsis severity is rooted in the intricate interplay between neutrophil and lymphocyte responses. In sepsis, the immune system’s initial response is characterized by neutrophil activation, leading to neutrophilia. This increase in neutrophil count is often accompanied by a reciprocal decrease in lymphocyte count, resulting in lymphopenia. Consequently, the NLR becomes elevated, reflecting the severity of the inflammatory response. While neutrophils are essential for combating infection, they also contribute to tissue damage and organ dysfunction through the release of reactive oxygen species and pro-inflammatory cytokines such as tumor necrosis factor-alpha and IL-6. These molecules exacerbate inflammation, leading to endothelial damage, increased vascular permeability, and ultimately organ failure. Conversely, lymphocytes play a pivotal role in resolving inflammation and promoting immune homeostasis, regulating the immune response by secreting anti-inflammatory cytokines such as IL-10 and facilitating pathogen clearance. An elevated NLR indicates a disrupted balance between these immune responses, with neutrophil-mediated inflammation overwhelming lymphocyte-driven resolution. This imbalance predicts worse outcomes, as an unchecked inflammatory response leads to tissue damage, organ dysfunction, and increased mortality. Furthermore, sustained lymphopenia compromises the host's ability to mount an adaptive immune response, rendering them more susceptible to secondary infections and perpetuating the cycle of sepsis [18-20].

Our study’s findings have significant implications for clinical practice. NLR can be utilized as a simple, inexpensive, and readily available biomarker to identify patients at high risk of developing severe sepsis or septic shock. Early recognition of these patients can facilitate timely interventions, ultimately improving outcomes. Additionally, NLR can monitor disease progression and response to treatment, enabling clinicians to adjust management strategies accordingly. Furthermore, our results suggest that NLR may serve as a useful tool for stratifying patients in clinical trials, thereby enhancing the accuracy of sepsis research.

However, our study does have several limitations. These include a relatively small sample size, a single-setting population, and a cross-sectional design. Furthermore, we did not explore NLR’s relationship with clinically meaningful outcomes such as mortality, length of ICU stay, and recovery. These limitations may restrict the general applicability of our findings. Therefore, future studies with larger sample sizes, diverse populations, different study designs, and outcome-based assessments are needed to validate the findings of the current study.

## Conclusions

This study has revealed a significant positive correlation between the NLR and sepsis severity, as measured by the SOFA scoring system. Higher NLR values were consistently linked to increased sepsis severity, and this direct relationship was confirmed through a simple linear regression model. These findings suggest that NLR is a valuable, efficient, and cost-effective predictor of sepsis severity in ICU patients. Regular monitoring of NLR is recommended to facilitate timely interventions and improve patient outcomes. Clinicians should consider NLR alongside other diagnostic tools for a comprehensive assessment of sepsis severity. By integrating NLR into clinical practice, there is potential to enhance patient outcomes and reduce mortality rates among sepsis patients. This study contributes to the growing body of evidence supporting the role of NLR in sepsis management and highlights the necessity of incorporating it into clinical guidelines and protocols.
